# Small Intestinal Microbiota Oscillations, Host Effects and Regulation—A Zoom into Three Key Effector Molecules

**DOI:** 10.3390/biology12010142

**Published:** 2023-01-16

**Authors:** Karina Ratiner, Tahel Fachler-Sharp, Eran Elinav

**Affiliations:** 1 Systems Immunology Department, Weisman Institute of Science, Rehovot 7610001, Israel; 2 Department of Dermatology, Hadassah-Hebrew University Medical Center, Jerusalem 9987500, Israel; 3 Microbiota & Cancer Division, Deutsches Krebsforschungszentrum (DKFZ), 69120 Heidelberg, Germany

**Keywords:** circadian clock, small intestine, microbiome, dietary timing, segmented filamentous bacteria

## Abstract

**Simple Summary:**

The gut microbiota and its secreted molecules feature a daily rhythm that interacts with the host and influences its function in health and disease. Immune-related molecules are involved in the daily interaction between the microbiota and the host and can be influenced by diet, including fasting and feeding cycles. In this review, we delve into the specific impacts of Reg3γ, IgA, and MHCII to showcase the varied effects of the gut microbiota's daily activity on the host. We also discuss current challenges, remaining questions, and perspectives in understanding the relationship between the microbiome and circadian rhythms.

**Abstract:**

The gut microbiota features a unique diurnal rhythmicity which contributes to modulation of host physiology and homeostasis. The composition and activity of the microbiota and its secreted molecules influence the intestinal milieu and neighboring organs, such as the liver. Multiple immune-related molecules have been linked to the diurnal microbiota-host interaction, including Reg3γ, IgA, and MHCII, which are secreted or expressed on the gut surface and directly interact with intestinal bacteria. These molecules are also strongly influenced by dietary patterns, such as high-fat diet and time-restricted feeding, which are already known to modulate microbial rhythms and peripheral clocks. Herein, we use Reg3γ, IgA, and MHCII as test cases to highlight the divergent effects mediated by the diurnal activity of the gut microbiota and their downstream host effects. We further highlight current challenges and conflicts, remaining questions, and perspectives toward a holistic understanding of the microbiome’s impacts on circadian human behavior.

## 1. Introduction

**Circadian activity:** Almost every aspect of life exhibits 24 h oscillations, ranging from cellular gene expression to behavior. These oscillations are generated by the host’s molecular clock, which is made up of several core genes that are expressed in most, if not all, mammalian tissues [1,2,3], including the brain [4] and the epithelial cells of the colon and **small intestine** (**SI**) [5,6,7,8,9]. The core clock genes include the transcriptional factors *BMAL1* and *CLOCK*, whose protein products dimerize, enter the nucleus, and activate the transcription of the repressor genes *PERs* and *CRYs* [10]. These factors also modulate the expression of many core clock genes and clock output genes, such as *REV-ERBs* and *RORs* [10]. It is believed that the various clocks found in the body are organized in a hierarchical manner, with the clock located in the **suprachiasmatic nucleus** (**SCN**) of the brain serving as the central clock that controls the clocks in peripheral organs [4]. This central clock receives input from the environment and regulates processes such as the sleep/wake cycle, hormone production, and rhythmic food intake [11,12]. In addition, the central clock sends signals to other parts of the body, known as peripheral clocks, to synchronize local processes such as metabolism [12]. In addition to SCN-clock regulation, external factors can also control peripheral clocks independently, particularly through feeding and fasting cycles that bypass central clock control [13]. In fact, the feeding/fasting cycle is recognized as one of the most powerful synchronizers of peripheral clocks and may have a significant impact on health [14], highlighting the importance of chrono-nutrition. It is worth mentioning that mice, which are nocturnal (awake at night and asleep during the day), tend to consume most of their food during the dark phase, while humans, who follow a diurnal pattern (awake and active during the day and asleep at night), eat during the light period [15,16]. As such, it is necessary to consider these differences in interpreting experiments involving nocturnal animals.

**Microbiota diurnal activity**: In recent years, numerous studies have shown that the microbiota fluctuates throughout the day [7,17,18,19,20,21,22,23,24]. Even though the identified taxonomy of oscillating bacteria may vary from study to study, the phenomenon of microbial rhythmicity remains remarkably consistent across studies and appears to be present in mice [7,17,18,19,20,21,22,23,24,25,26,27,28,29,30], rats [31], meerkats [32], humans [25,33,34,35,36], and non-mammals (such as chickens [37] and fish [38,39]). In 2014, it was discovered that the gut microbiota undergoes diurnal oscillations in humans and mice [25]. ***Lactobacillus reuteri****(**L. reuteri**)* and *Dehalobacterium* spp., for instance, exhibit oscillatory abundances in murine feces [25]. These microbial oscillations were perturbed by jetlag, **high-fat diet** (**HFD**), or genetic mutation of the core clock genes *Per1* and *Per2* (*Per1/2* knock-out mice) [17,25]. In mice, subjecting any of these three perturbation models to a **time-restricted feeding** (**TRF**) protocol can restore these microbial oscillations [25]. This finding suggests that the host’s feeding rhythms, which are driven by its circadian clock, may play a role in driving microbial oscillations. Interestingly, transcriptomics analysis of the colon and liver of either antibiotic-treated or germ-free mice reveals that the classical clock genes are still rhythmic in the absence of microbes [7,8]. Therefore, the hierarchical control of host rhythms by microbiota seems to be mediated by the circadian clock and feeding rhythms.

Diurnal variations in gut microbes occur in the luminal and mucosal compartments at the level of composition and function [7,25]. The dark phase is characterized by the maximum microbiota activity related to energy metabolism, peptidoglycan synthesis, DNA repair, and mucus degradation [25], while the light phase involves the maximum activity of detoxification, flagellar assembly, and chemotaxis [25]. Further research into the colonic spatial differences reveals that some of these functions are phase-opposite in the mucosal compartment, particularly chemotaxis and flagellar assembly, which are more prevalent during the dark phase at ZT18 [7]. During this time period, the greatest number of bacteria colonize the mucosal epithelium [7]. The observations of daily fluctuations in microbial adherence suggest a link to the host’s circadian clock. Indeed, *Per1/2* knock-out mice feature disruption of mucosal-associated bacterial rhythms [7]. It appears that these core clock genes, *Per1* and *Per2*, do not directly govern this phenomenon but rather influence it via feeding rhythms. Under standard light/dark cycles and free-feeding conditions, wild-type mice exhibit robust rhythmicity of daily food intake, but this pattern is diminished in *Per1/2* knock-out mice and is absent in mice housed in constant darkness [40]. Although maintaining rhythmic eating requires the expression of *Per1* and *Per2* genes, these rhythms can be induced externally by restricting food access to certain times of the day [40]. When *Per1/2* knock-out mice are fed only for a few hours a day, the mucosal-associated bacteria adhering to the colon resume their rhythms [7]. In turn, the rhythmicity of mucosal-associated bacteria is responsible for the host’s metabolomic, transcriptomic, and epigenetic oscillations, which ultimately affects lipid absorption and liver toxicity [7,41].

**Small intestinal microbiota diurnal activity**: Most of the above initial circadian microbiota discoveries were made in the colon (using fecal samples as proxies for colonic microbiota configuration), yielding valuable insights into the nature of microbiota oscillations. However, the SI, representing most of the mammalian gut, and the site in which most metabolic, immune interactions, and food digestion take place, has remained understudied. Recent studies have identified unique compositional and functional features of SI microbiota diurnal activities, their regulation by diet, interactions with local immune and metabolic host hubs, and diurnally-shifting secretion of microbiota-modulated metabolites, all influencing key local activities in this organ. For example, **segmented filamentous bacteria** (**SFBs**), a Gram-positive bacterium that can adhere to intestinal epithelial cells [7], can be most abundantly found in the terminal ileum of mice [42]. The SFBs, also known as *Candidatus* Arthromitus or *Candidatus* Savagella, are characterized by their long filamentous structure and while morphologically similar between species, SFB populations differ genetically [43,44,45,46]. Due to these genetic variations, SFBs adhere to intestinal epithelial cells in a host-specific manner, making them cross-species incompatible in this regard [43,44]. In germ-free mice mono-colonized with either mouse or rat SFBs, only mouse-derived SFBs adhere to intestinal epithelial cells and exhibit similar rhythms to normal **specific pathogen-free** (**SPF**) mice, in terms of RNA transcripts [7]. Without diurnal attachment of SFBs, intestinal epithelial cells and hepatocytes exhibit perturbed oscillations in transcriptome, epigenome, and detoxification reactions [7]. Of note, although the requirement of bacterial attachment to host rhythm was established in SFBs [7], other mucosal-associated bacteria could also affect these rhythms.

In addition to the role in modulating circadian rhythms, SFB attachment is critical for maintaining several functions of the SI immune system in mice, including T-cell maturation and differentiation and IgA production [47,48,49]. Because SFBs are attached to the terminal ileum mucosa, obtaining intestinal biopsy samples, which is a difficult and invasive procedure, is necessary for their study. Therefore, in comparison to mice, there are far fewer studies mechanistically focusing on human SFBs. Despite inconsistent evidence from studies [50], some reports suggest that SFBs colonize the human SI may induce IgA secretion and T-cell maturation while featuring some distinct genetic characteristics from mouse SFB [43,51]. The noted murine commensal impacts on host circadian rhythmicity, antimicrobial peptide secretion, immunoglobulin production, and MHCII expression merit future studies in the human setting.

**Figure 1:**
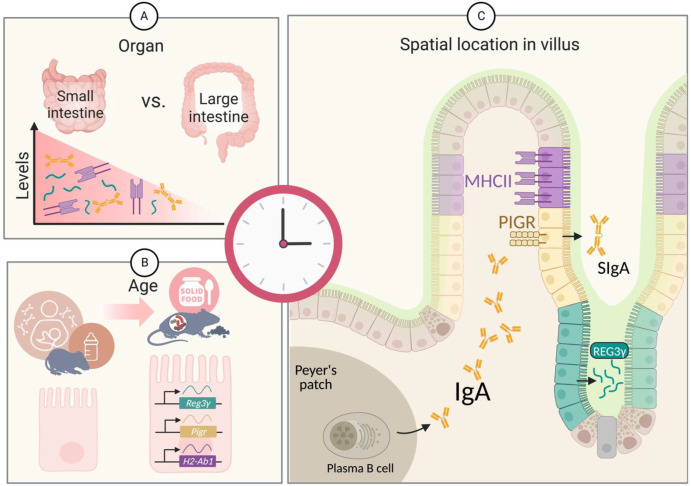
**Factors influencing genes and proteins related to REG3γ, IgA, and MHCII in the gastrointestinal tract.** (**A**) The three molecules are more abundant in the SI than in the large intestine. (**B**) During early infancy, production of the three molecules remains low, with the source of IgA mainly coming from breast milk. With the onset of solid food intake, an infant’s body begins to produce IgA, and the genes encoding *Reg3γ*, *Pigr* (IgA transporter), and *H2-Ab1* (MHCII) are gradually expressed along the SI. (**C**) *Reg3γ*-expressing SI epithelial cells are located at the bottom of villi, while MHCII-expressing epithelial cells are located upper part of villi. The *Pigr*-expressing cells (yellow), which can secrete the IgA produced by plasma B cells into the lumen, are located in the middle of villi. Figure created with BioRender (biorender.com).

In this review, we will exemplify the circadian host/microbiota cross-regulatory concept by focusing on three of the most widely studied molecules in this context, Reg3γ, IgA, and MHCII. All three host molecules are components of the innate immune system which are produced by multiple host cells, with the highest levels noted in the SI (Figure 1A), where they interact directly with the microbiota and digested food particles. Interestingly, diet, which plays a role in synchronizing circadian rhythms, influences the activity and production of these three molecules. Recent single-cell RNA-seq analysis showed that mouse SI epithelial cells express genes associated with these molecules in an age-dependent manner, possibly due to changes in microbiota colonization and eating patterns [52]. From the age of 3 to 6 weeks, the expression of genes encoding the MHCII (*H2-Ab1*), secretory IgA receptor (*Pigr*), and antimicrobial peptides of the Reg3 family (including *Reg3γ*) begins to rise [52] (Figure 1B). During these age periods, mice start consuming solid food, and their immune system and microbiota are stabilized [52]. The location of these genes along the SI villi varies. SI epithelial cells expressing *Reg3γ* are located near the bottom of villi, *Pigr* expressing cells are located in the middle of villi, while H2-Ab1 is mostly located in the upper part of SI villi [53,54] (Figure 1C). As elaborated below, recent studies have demonstrated that REG3γ, IgA, and MHCII demonstrate temporal variations which are extensively modulated by diet and the microbiota, which collectively bear direct consequences on host physiology and risk of disease.

## 2. The Antimicrobial Peptide REG3γ

**Regenerating islet-derived protein III gamma** (**REG3γ**) is a secreted antimicrobial peptide that constitutes an integral part of the mammalian antimicrobial defense. Reg3γ is expressed in SI enterocytes and Paneth cells in response to the activation of **toll-like receptors** (**TLRs**) by microorganism-associated molecular patterns [55,56]. Reg3γ binds to the peptidoglycan layer on the surface of Gram-positive pathogens and eliminates them by creating pores in their membranes [57]. Under normal conditions, Reg3γ reduces the adhesion of mucosal bacteria to intestinal epithelial cells [58]. Of note, the adhesion of microbes to intestinal epithelial cells is also controlled by daily rhythms [7] (see Section 1). In the colon, *Reg3γ*-deficient mice exhibit constant elevation and disrupted diurnal rhythms in the numbers of microbes adhering to the mucus layer [7]. Since the rhythmic attachment of mucosal-associated bacteria leads to the global programming of host functional oscillations [7], the absence of *Reg3γ* may likewise disrupt host rhythmic function, meriting further studies. *Reg3γ* expression is low in the colon and more pronounced in the SI [59,60]. At the bottom of the villi, SI epithelial cells express high levels of Reg3γ (Figure 1C) [53,61]. Several studies have also reported Reg3γ oscillations in the ileum [9,26,27], but the exact pattern of mRNA or protein expression varies between them—in one, the expression of the Reg3γ gene is noted to be reduced near the middle of the light phase and at its lowest around ZT12 [9], but two recent studies indicate an opposite pattern [26,27], in which Reg3γ expression is increased at the beginning of the light phase and reached its maximum expression around ZT10 [26] and ZT12 [27]. Both studies confirmed this oscillation at the protein level, using Western blot and immunostaining of REG3γ, collectively suggesting that its levels increased during the light phase [26,27].

**Reg3γ regulation by SFB and dietary timing.** The diurnal pattern of Reg3γ expression in the ileum of SPF mice [9,26,27] is compromised in antibiotic-treated [9] or germ-free [27] mice. In both microbiota-disrupted conditions, the levels of Reg3γ remain low throughout the course of the day [9,27]. Based on an earlier study in which Thaiss et al. [7] revealed that Reg3γ is necessary for sustaining the diurnal adherence of mucosal-associated bacteria, Brooks et al. [27] investigated it in the upper gastrointestinal tract and found that the total density of SI mucosal-associated bacteria, excluding SFBs, correlated inversely with the daily expression of Reg3γ [27]. Unlike total mucosal-associated bacteria, the adhesions and levels of SFBs were correlated with Reg3γ expression with both increasing at the end of the light hours [27], indicating that Reg3γ likely does not inhibit SFBs. In the SI of *Reg3γ*-deficient mice, the diurnal SFB attachment pattern remained intact. In contrast, densities of other mucosa-associated bacteria are arrhythmic in *Reg3γ*-deficient mice, suggesting that *Reg3γ* may regulate diurnal rhythms of non-SFB bacteria that colonize the SI mucosa [27]. Inversely, mono-colonization of germ-free mice with SFBs is able to restore levels of REG3γ at ZT12, suggesting that SFBs impact Reg3γ expression and oscillations.

**Figure 2:**
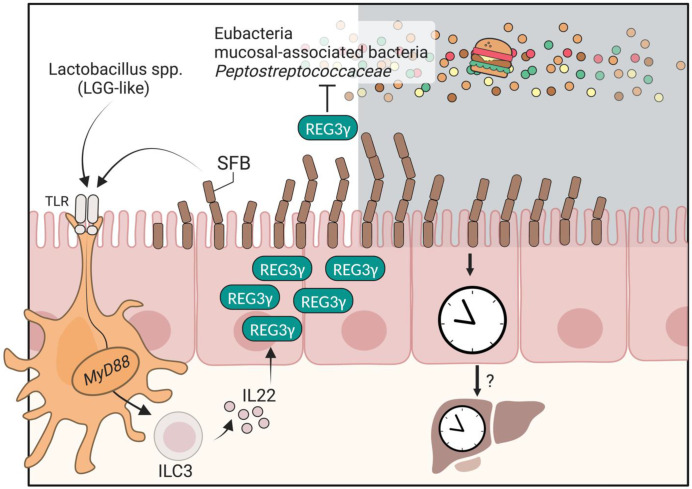
**Diurnal regulation of small intestinal REG3γ secretion.** In response to feeding rhythms, SFB and Lactobacillus spp activate TLR-MyD88-IL22 pathways, thereby leading to REG3γ secretion by SI epithelial cells. In turn, REG3γ inhibits the expansion of luminal microbes and mucosal-associated bacteria. Diurnal consumption of food and microbial oscillations, in turn, influence the diurnal activity of the host, such as liver rhythmicity. LGG, *Lactobacillus rhamnosus* GG; TLR, toll-like receptor; ILC3, type-3 innate lymphoid cells; IL22, interleukin-22; SFB, segmented filamentous bacteria. Figure created with BioRender (biorender.com).

Interestingly, upon rhythmic attachment of SFB, myeloid cells are activated by the TLR-MyD88 axis and secrete **Interleukin-23** (**IL-23**), which induces **ILC3** (**type-3 innate lymphoid cells**) to secrete another cytokine, IL-22 (Figure 2). Then, IL-22 drives rhythmic activation of **STAT3** (**signal transducer and activator of transcription 3**) activation and phosphorylation in intestinal epithelial cells. Activated STAT3 induces the transcript of antimicrobial peptides, among them *Reg3γ*. In turn, elevated antimicrobial peptides suppress the attachment of mucosal-associated bacteria to the SI epithelium (Figure 2) [27]. A physiological consequence of these antimicrobial peptide oscillations is a day/night difference in resistance levels to infection [27].

Several studies have shown that clock-controlled feeding rhythms constitute the main driver of rhythmic intestinal microbiota patterns [7,54]. Furthermore, mono-colonization with SFB generates diurnal rhythms in the germ-free host [7,54]. Importantly, *Clock^Δ19/^^Δ19^* mice featuring a perturbated feeding rhythms feature an arrhythmic Reg3γ [27] (Table 1). Furthermore, SFB attachment is regulated by feeding and fasting regimens [7,26,62]. Considering that mice are nocturnal and consume most of their food in the dark, these findings suggest that temporal fasting may induce Reg3γ expression. Indeed, when mice are subjected to forced day or night feeding, REG3γ reaches its maximum level during the fasting period in each setup [27]. This does not necessarily imply that exposure to food initiates REG3γ activation, as fasting may also trigger REG3γ activation [62]. However, when mice are subjected to 24 h fasting, the levels of REG3γ protein remain low at all time points [27]. Additional studies are needed to fully unravel the effects of dietary timing on REG3γ rhythmicity. Frazier et al. [26] has shown that the ileal microbiota of mice produces a diurnal expression of the protein Reg3γ in response to a normal diet but not in response to an HFD. Importantly, only regular diet-fed SPF mice exhibited oscillations in Reg3γ expression. Lactobacillus and Reg3γ were positively correlated, and *Clostridiaceae* and *Peptostreptococcaceae* were negatively correlated to this circadian feature. Induction of Reg3γ by ***Lactobacillus rhamnosus* GG** (**LGG**) was dependent on MyD88 signaling, while exogenous in vitro treatment with recombinant REG3γ could inhibit the growth of certain strains of bacteria (Figure 2). While both studies suggest that Reg3γ plays an important role in food- and microbial-regulated circadian host/microbiota interactions, the full commensal repertoire, respective functions, and host circuits responsible for the regulation of Reg3γ merit future studies.

## 3. The Secreted Antibody IgA

Another key molecule participating in the diurnal host/microbiota interaction is **Immunoglobulin A** (**IgA**). IgA, or its secreted form, **secretory IgA**, is produced by plasma B cells in mucosal membranes. As a result, IgA antibodies are highly prevalent in mucosal secretions, including human breast milk, saliva, tears, sweat, and intestinal fluid [63,64]. In these niches, secretory IgA plays a protective role by preventing pathogens from adhering and invasion as well as by interacting with beneficial commensal microbes [65]. Studies in humans and rodents have demonstrated that secretory IgA fluctuates diurnally in nasal secretions [66,67,68,69], breast milk [70,71], saliva [72,73,74,75], feces, and the intestinal lumen [21,76,77]. At ZT6, around the middle of the light period, mice feature the highest levels of secretory IgA in their SI and feces, and these levels are reduced at the end of the light period [21,76]. The underlying cause of the rhythmic secretion of IgA is not completely clear, and it may differ from one region of the body to another.

Most plasma B cells in humans and mice are located in the intestinal mucosa, where they secrete large quantities (grams) of dimeric IgA daily, even in the absence of classical pathogens [78,79]. In fact, there is a bi-directional interaction between IgA responses and microbiota, in which IgA can target the commensal species living in the gastrointestinal tract but also requires these microbes to function properly. IgA transport to the intestinal lumen is facilitated by the **polymeric immunoglobulin receptor** (**pIgR**) expressed on intestinal epithelial cells. This receptor recognizes IgA produced by plasma B cells and transfers it into the intestinal lumen, where it binds bacteria. The *pIgR*-expressing intestinal epithelial cells are located close to the bottom of the villi, near the Reg3γ-producing enterocytes (Figure 1), which are also known for their capacity to neutralize bacteria [53]. Unlike Reg3γ, IgA is capable of specifically binding to distinct microorganisms. The physical interactions between microbes and IgA have important influences on host health and survival [58].

At early stages of life, intestinal mucosal IgA diurnal rhythms are delivered through breast milk colostrum [80]. The delivered secretory IgA influences infant health, for example by preventing necrotizing enterocolitis [81]. Increased taxonomic variability in early weaned animal models has been described with decreased concentrations of secretory IgA [82]. After weaning from breastfeeding, IgA is autonomously and diurnally produced. Any of the factors involved in the production and intestinal delivery of IgA may contribute to its rhythmicity, including rhythmic production by plasma B cells, diurnal expression of pIgR, or rhythmic secretion of CCL25, a chemokine that attracts IgA-producing cells [53,83]. However, recent studies suggest there is no indication of oscillatory expression of the *Pigr* or *Ccl25* genes in the SI [20,21]. Several genes involved in metabolism and antibody secretion in SI IgA^+^ plasma B cells exhibit diurnal rhythms [21]. Penny et al. [21] demonstrated that IgA rhythmicity is largely influenced by dietary patterns and microbial signals rather than by intrinsic core clock activity. In mice deficient in the core clock gene *Bmal1* specifically in plasma cells or intestinal epithelial cells, IgA secretion remained rhythmic with maximal IgA concentrations noted at around ZT6. Conversely, induction of an inverted TRF feeding led to a concomitant inversion of the phase of IgA level among mice that were restricted to daytime feeding. HFD similarly disrupted IgA rhythmicity after 6 weeks, accompanied by a significant increase in fecal IgA.

**Figure 3:**
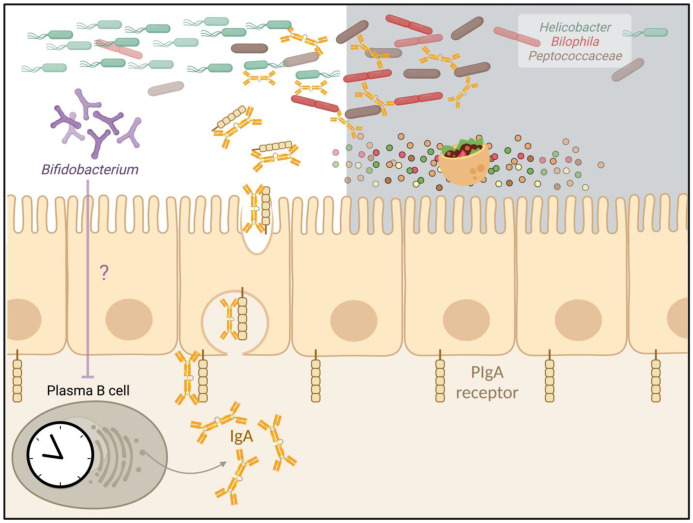
**Diurnal oscillations of secretory IgA.** Plasma B cells diurnally secrete IgA, which is transferred through the intestinal epithelial cells via PIgA receptor. In turn, secretory IgA binds to commensal microbes and can modulate their rhythmicity. In addition, IgA secretion might be regulated by the microbiota. For example, such secretion may be suppressed by Bifidobacterium. Figure created with BioRender (biorender.com).

**IgA regulation by the microbiota.** Antibiotic-treated mice developed an increase in fecal IgA [21]. Conversely, mice perturbed of IgA-producing cells developed marked alterations in microbial composition and functional oscillations, indicating possible mutual feedback. Sequencing of IgA-bound versus unbound bacteria (referred as to IgA-seq) in non-IgA perturbed mice demonstrated that IgA-bound microbes were oscillating [21], while many IgA-bound bacteria lost their rhythmicity in IgA-deficient mice, including *Helicobacter*, *Bilophila*, and *Peptococcaceae*. Collectively, these results suggested that secretory IgA controls specific members of oscillating microbes through direct interactions (Figure 3) [21]. Using germ-free mice mono-colonized with *E. coli*, several mechanisms were suggested by which microbial surface IgA binding regulates the microbiota [84]. These include reduced bacterial motility and protection from the toxic effects of bile acids, through mechanisms whose antigenic specificity remains unclear [84]. Of note, SFB mono-colonization markedly increases mucosal IgA levels in mice [85]. In humans, lower counts of *Bifidobacterium* encountered significantly higher IgA levels in IgA nephropathy patients [86]. Whether SFB, *Bifidobacterium,* or other members of the microbiota leading to increased IgA production can modulate immunity and immunopathology remains elusive to date.

## 4. The Antigen Presentation Molecule MHC Class II

**Figure 4:**
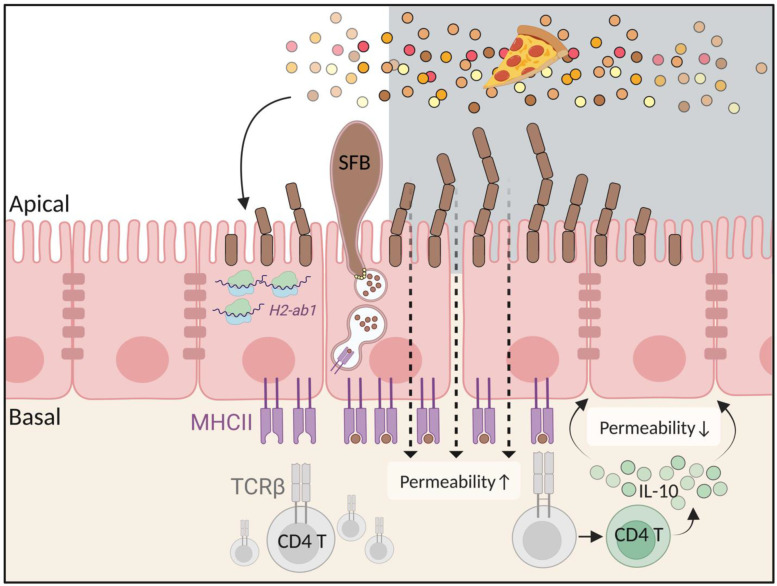
**MHCII diurnal expression by intestinal epithelial cells.** The diurnal rhythm of MHCII is noted both at the mRNA and protein level. MHCII expression gradually increases during the inactive phase. During the active phase and associated exposure to food, the MHCII receptor on intestinal epithelial cells, presenting commensal antigens, engages and activates IL-10-producing CD4^+^ T cells (Tr1 cells), which, in turn, modulate gut permeability in a circadian manner. TCR, T-cell receptor; MHC, major histocompatibility complex class II; IL10, interleukin-10; SFB, segmented filamentous bacteria. Figure created with BioRender (biorender.com).

**Major histocompatibility complex class II** (**MHCII**) participates in the diurnal interaction between the host and the gut microbiota [54]. MHCII is a surface receptor that displays antigens originating in the external environment to T-cell receptors expressed on CD4^+^ T cells, resulting in antigen-specific immune activation [87,88]. Although MHCII is predominantly expressed on the surface of ‘professional’ antigen-presenting cells, such as dendritic cells and B cells, it is also expressed on certain non-hematopoietic intestinal epithelial cells (the latter collectively termed ‘non-professional’ antigen-presenting cells [87,88]). Compared to the murine cecum and colon, MHCII levels are significantly higher in the duodenum, jejunum, and ileum [54,89]. In these regions, the MHCII surface protein exhibits diurnal oscillations at the basal side of epithelial cells (Figure 4), with maximal levels noted around ZT10 [54]. The rhythmicity of MHCII protein is phase-shifted in *Per1/2* knock-out mice and completely abrogated in jet-lagged mice (Table 1), suggesting a close relationship to the host clock and clock-driven behaviors, such as feeding patterns [54]. MHCII rhythmicity is also present at the mRNA level, but this oscillation takes place in the apical rather than basal intracellular region of epithelial cells, with the highest levels noted at around ZT8 (Figure 4) [54]. Under HFD, MHCII levels remain non-fluctuating and low throughout the day, while a TRF can restore rhythmicity in arrhythmic mouse models (such as in jet-lagged or genetically mutated mice) [54]. Therefore, it is likely that feeding-fasting cycles, rather than diet composition per se, shape the diurnal rhythms of SI MHCII [54].

**MHCII regulation by the microbiota.** Antibiotic-treated or germ-free mice feature a substantial reduction and loss oscillation of MHCII [54]. Furthermore, germ-free mice transplanted with the microbiota from SPF donors on a HFD feature lower levels of MHCII compared to recipients of the microbiota from regular-diet-fed donors, even though they had not been directly exposed to these diets [54]. Collectively, these results suggest that microbial alternations in response to dietary configurations, and not the diet itself, may modulate MHCII. Using cross-correlation analysis of 16S rDNA sequencing, several microbes that may modulate MHCII were characterized, including SFBs, *Lachnospiraceae*, and *Akkermansia*, were suggested as MHCII inducers, while *Lactobacillus*, *Sutterella*, *Erwinia*, *Allobaculum*, and *Ruminococcus* were suggested as suppressing MHCII [54]. Indeed, colonization of SFB into germ-free mice induced an increase in MHCII. To test if SFB gut mucosal attachment was necessary for MHCII induction, either mouse or rat SFB (with only the former adhering to the mouse gut mucosa) were mono-colonized into germ-free mice. Indeed, only mouse SFB induced MHCII induction, suggesting that SFB attachment is critical to MHCII expansion [54], likely through endocytosis-dependent pathways ([90,91] and Figure 4) similar to antigen processing and presentation on MHCII [64]. Indeed, the disruption of several molecules involved in SFB endocytosis, such as serum amyloid A or CDC42, disrupted the diurnal rhythmicity of MHCII [54].

**Diurnal SI epithelial MHCII functions.** One major consequence of diurnal MHCII expression is related to the regulation of intestinal permeability. Under normal conditions, intestinal permeability gradually increases during the mid-day hours and peaks as the dark period begins around ZT16, shortly after MHCII levels peak (Figure 4). In mice that lack MHCII expression in their intestinal epithelial cells, a non-fluctuating increase is noted in the permeability of the intestinal barrier [54]. Intestinal epithelial circadian MHCII expression may be mediated by diurnal induction of intra-epithelial CD4 T cells that produce the anti-inflammatory cytokine IL-10, which impacts SI trans- and para-cellular permeability [54] (Figure 4). This rhythmicity is abrogated when the diurnal diet-microbiota-MHCII axis is disrupted [54]. Excessive intestinal permeability allows luminal contents to influx into the host and triggers an uncontrolled inflammatory response. Indeed, MHCII reductions by an HFD or complete lack of MHCII in intestinal epithelial cells cause hyperpermeability and exacerbation of SI enteritis mimicking Crohn’s disease in mice [54]. Additional research, particularly in human subjects, is necessary in fully decoding the clinical implications of these observations in the human setting.

A recent pre-print suggests that colonization with SFB induces the differentiation of CD4^+^ T cells carrying TCRs that specifically recognize SFB-loaded MHCII into granzyme-expressing cells, which modulate epithelial cell turnover [52]. Some intestinal stem cells may also express MHCII, albeit at lower concentrations [92,93]. While intestinal stem cells follow circadian rhythms in their differentiation and cell cycling [94], they do not necessarily harbor the diurnal MHCII phenotype described in SI intestinal epithelial cells, necessitating future exploration. Interestingly, as in intestinal epithelial cells, HFD suppresses MHCII levels in intestinal stem cells [54,93]. Of note, some of the microbes modulating SI epithelial MHCII, such as *Helicobacteraceae*, vary across studies and are suggested to repress MHCII in epithelial cells [54] and induce it in stem cells [93]. Microbial diurnal impacts on SI epithelial stem cells will likely constitute exciting avenues of future research.

MHCII may also influence B cells, which could in turn impact the microbiota [95]. Single-cell RNA comparison of sorted immune cells from animals lacking MHCII in intestinal epithelial cells and wild-type mice revealed changes in B-cell receptor repertoires. The loss of MHCII in intestinal epithelial cells resulted in reduced affinity of secretory IgA to intestinal microbes [95]. Consequently, mice with intestinal epithelial cells lacking MHCII featured fewer IgA-bound gut bacteria [95], increased inter-individual variation in the ileal microbiota, coupled with an expansion of the S24-7 family and reduction of *Bifidobacterium pseudolongum* [95]. This result, however, somewhat contradicts previous research indicating that the microbiota may act as an upstream regulator of MHCII [54]. Hence, further exploration of the hierarchical relationship between MHCII and the microbiota is necessary. Moreover, given the diurnal variation of secretory IgA [21], further research is needed to explore the putative intersection of the MHCII-IgA diurnal axes. Collectively, diurnally oscillating SI MHCII is involved in anti-inflammatory cytokine secretion [54], regulation of gut permeability [54], epithelial turnover [52], cellular proliferation [52], and determination of IgA repertoires [95]. This diurnal expression may be modulated by diet and the microbiota, and impact downstream diseases such as SI Crohn’s disease and cancer. These merit further studies.

**Table 1:** Diurnal features of REG3γ, IgA, and MHCII (all data refer to mice).

		REG3γ	IgA	MHCII
Oscillations (steady-state)		Mostly reported as higher at ~ZT12 [26,27]	Fecal and small intestinal IgA are higher at ZT6 [21,76]	H2-Ab1 (MHCII mRNA) peak ~ZT8Surface protein ~ZT10-ZT12 [20,54]
Influencing factors	Circadian	▪ Rev-erbα^−/−^ mice: normal Reg3γ rhythms ▪ Clock^Δ19/19^ mice: loss of Reg3γ oscillations [27]	▪ Mb1^Cre^ x Bmal1^fl/fl^ mice: fecal IgA is rhythmic [21] ▪ Villin^Cre^ x Bmal1^fl/fl^ mice: fecal IgA is rhythmic [21]	▪ Per1/2 knock-out mice: Phase shift of MHCII [54] ▪ Villin^Cre^ x Bmal1^fl/fl^ mice: MHCII is still rhythmic [54] ▪ Jet-lag: arrhythmic MHCII [54]
		Note: Rev-erbα^-/-^ mice exhibit normal eating rhythms, Clock^Δ19/19^ mice are arrhythmic, whereas Per1/2 knock-out mice show perturbated rhythmic eating. Mb1-Cre for plasma and B cells and Villin-Cre for intestinal epithelial cells.		
	Diet	▪ HFD: Reg3γ is arrhythmic and constantly low [20,26] ▪ Inverted feeding (Day-TRF): Reg3γ shows inverted rhythms [27] ▪ 12-h fasting: Reg3γ is higher compared to fed mice [62] ▪ 24-h fasting: Reg3γ is constantly low [27].	▪ HFD: fecal IgA is arrhythmic and constantly high [21] ▪ Inverted feeding (Day-TRF): fecal IgA show inverted rhythms and reduced amplitude [21]	▪ HFD: MHCII is arrhythmic and constantly low [54] ▪ Inverted feeding (Day-TRF): Phase shift of MHCII [54]
	Microbiota	▪ Antibiotics: Reg3γ is constantly low [9] ▪ Germ-free: Reg3γ is constantly low [26,27]. ▪ Germ-free mono-colonized with SFB: Reg3γ is higher at ZT12 [27] ▪ Enteroids treated with LGG: Reg3γ is elevated [26]	▪ Antibiotics: fecal IgA is constantly high [21]	▪ Germ-free and antibiotics: MHCII is constantly low [54] ▪ Germ-free mice mono-colonized with mouse SFB: MHCII is elevated [54]
Function		▪ Elimination of pathogens, implicated in susceptibility to infections [27]. ▪ Control of commensal microbes, implicated in glycemic response [26]	▪ Elimination of specific microbes, implicated in necrotizing enterocolitis (infants) [81]	▪ Permeability of the gut, implicated in inflammatory bowel disease [54]

## 5. Challenges and Limitations

Research on host/microbiota circadian interactions has identified key molecules, including Reg3γ, IgA, and MHCII, whose oscillatory behaviors in the SI may impact multiple health and disease processes. Oscillations and total levels of these molecules are sensitive to perturbations in circadian rhythm, nutrition, and microbiota (see Table 1), suggesting that common mechanisms may regulate their diurnal behavior and downstream effector functions, which may result in cross-regulation between the molecules. For example, MHCII is known to influence IgA specificity [21], while *Reg3γ*-deficient mice feature elevated IgA-producing cells in the SI lamina propria and increased fecal levels of IgA compared with wild-type mice [59]. Such cross-regulatory networks merit future studies.

In expanding the scope of circadian host/microbiota research, several noted discrepancies between studies need to be clarified and technological and conceptual barriers overcome. These may result from methodological differences, confounding factors, and varied microbiota configurations in different vivaria. For example, different microbes including SFB [27] or *Lactobacillus* [26] have been suggested to regulate Reg3γ, likely depending on their luminal or mucosal locality. *Helicobacteraceae* were described as repressors [54], but also inducers [93], of MHCII in different subsets of MHCII-expressing epithelial cell types. Some of these differences may stem from the sequencing method used in identifying the microbiota in each study (e.g., 16S amplicon sequence of V1-V2 region of fecal samples [93] versus V4 region of the mucosal microbiota [54]). Variability in microbial identification may also be attributable to diverse sample sites (e.g., feces [93], colon [7], or SI [26,27]), differing regions of the SI (e.g., jejunum [54] vs. ileum [95]), or varied cell populations within the same intestinal region (intestinal epithelial cells [54] vs. stem cells [93]), or alternatively to differences in the prevailing commensal microbiota residing in different vivaria.

Different methods for quantifying REG3γ, IgA, and MHCII could likewise influence experimental conclusions. For example, the MHCII protein is a receptor that can be studied with FACS for surface fraction, Western blot for bulk levels, or immunostaining that can indicate its spatial localization. REG3γ, on the other hand, is a secretory peptide that can be quantified either intracellularly or extracellularly. Moreover, REG3γ and MHCII are differentially expressed by several cell populations, including Paneth cells, stem cells, immune cells, and intestinal epithelial cells. Of note, Reg3γ, IgA, and MHCII have been independently studied in the context of certain bacterial species, such as SFB and others, which can induce differential effects on each of the molecules [7,27,52,54,85]. Additionally, the effects of these bacteria can be influenced by various dietary and environmental factors [7]. To determine more precise influences of dietary content and timing on SFB, it is necessary to study the interactions between short-term and long-term fasting periods while assessing the impacts of different dietary macronutrients. Differences in microbial presence between vivaria can impact such regulatory effects. SFB, for example [27], is differentially present in mice derived from different vendors [48] and may be diurnally altered in a sex-specific manner [29]. Such gender differences [96,97,98,99] may generate “cage effects” in which differentially housed female and male mice result in a inter-cage gut microbiota drift. Such differences may be minimized by bedding exchange [8,99,100,101] and by the inclusion of both genders in key experimental repeats.

Human experimentation is likewise underperformed in circadian microbiome studies, preventing a more complete and causal understanding of the role of the microbiome in the diurnal regulation of various molecules and modulation by dietary timing. Indeed, most of the findings depicted in this review are based on animal studies. This is particularly concerning given the fact that translation of animal studies into the human setting remains challenging at times. For example, while animal studies have shown promising results in utilizing TRF to promote health benefits, human studies have not consistently reproduced the same findings [14]. Several factors may contribute to this translational difficulty, including human intra-individual variability in the microbiota and its response to dietary timing and content and the lack of invasive human sampling directly assessing the gastrointestinal microbiota configuration [14]. Further research is necessary to fully elucidate the role of bacterial rhythmicity on human physiology.

## 6. Conclusions

Diurnal host/microbiota interactions constitute a critical mechanism allowing human adaptations to their environment. This time-specific interactome is in part achieved by a diurnal secretion of the antimicrobial peptide Reg3γ, time-of-day-dependent immune regulation via MHCII-expressing enterocytes, and time-specific binding of secretory IgA to commensal microbes. Decoding the causal impacts of these and other molecules and pathways on human health and on microbiota-associated disease may enable harnessing the findings towards preventive and therapeutic interventions.

## Data Availability

Not applicable.
